# Psychotherapy Within Occupational Therapy Literature: A Scoping Review

**DOI:** 10.1177/00084174221102732

**Published:** 2022-07-26

**Authors:** Carrie Anne Marshall, Michelle Murphy, Kristina Marchiori, Suliman Aryobi, Pam Wener, Catherine White, Nadine Larivière, Roxanne Isard, Avneet Chohan, Mary Forhan, Niki Kiepek, Skye Barbic, Victoria Sarunsky, Sandra Moll

**Keywords:** Counselling, Group therapy, Humanism, Mental health, Psychodynamic, Counselling, humanisme, psychodynamique, santé mentale, thérapie de groupe

## Abstract

**Background.** Recent changes in the Canadian regulatory landscape have prompted reflections on the role and scope of occupational therapy in the provision of psychotherapy. **Purpose.** To document how psychotherapy has been explored in occupational therapy literature. **Method.** We conducted a scoping review following Preferred Reporting Items for Systematic Reviews and Meta-Analyses Scoping Review (PRISMA-ScR) guidelines by searching eight databases (e.g., Medline, AMED, CINAHL, EMBASE, PsycINFO, Cochrane Database of Systematic Reviews, Sociological Abstracts, and ProQuest Dissertations & Theses). Articles included at the full-text stage were subjected to a narrative synthesis. **Findings.** A total of 207 articles met the criteria for inclusion, spanning 93 years. 47.3% of these articles represented non-empirical literature, with only 14% representing effectiveness studies, suggesting that this body of literature remains in an early stage of development. **Implications.** Occupational therapists have been writing about and practicing psychotherapy for nearly a century, yet there remains an important opportunity to develop and evaluate occupation-based psychotherapy approaches. Effectiveness studies are needed.

## Résumé

**Description**. Des changements récents dans le paysage réglementaire canadien ont donné lieu à des réflexions sur le rôle et la portée de l'ergothérapie dans la prestation de services de psychothérapie. **But.** Documenter la manière dont la psychothérapie a été explorée dans la littérature sur l'ergothérapie. **Méthodologie.** Nous avons effectué une étude de portée en suivant les lignes directrices PRISMA-ScR et en interrogeant huit bases de données (Medline, AMED, CINAHL, EMBASE, PsycINFO, Cochrane Database of Systematic Reviews, Sociological Abstracts et Proquest Dissertations & Theses). Le texte intégral des articles retenus a fait l'objet d'une synthèse narrative. **Résultats.** 207 articles couvrant une période de 93 ans répondaient aux critères d'inclusion. 47,3 % de ces articles étaient non empiriques, et seulement 14 % étaient des études d'efficacité, ce qui suggère que ce corpus reste à un stade précoce de développement. **Conséquences.** Les ergothérapeutes écrivent sur la psychothérapie et la pratiquent depuis près d'un siècle. Pourtant, il reste des occasions importantes de développer et d'évaluer des approches de psychothérapie basées sur l'occupation. Des études d'efficacité sont nécessaires

## Introduction

According to the National Institutes of Health (NIH), psychotherapy is: “a term for a variety of treatment techniques that aim to help a person identify and change troubling emotions, thoughts, and behaviour” (NIH, 2021). Psychotherapy is variously defined and includes a range of approaches including talk-based therapies such as cognitive behavioural therapy (CBT), and activity-based approaches involving the use of play, art, and writing to help individuals attain mental well-being (Coles & Elliot, 2020; Cooper & Davis, 2016; Green et al., 2005; Copley et al., 1987). Occupational therapists working in mental health use a range of strategies to support individuals who are living with mental illness to function and participate in the activities that are meaningful to them in their daily lives (Krupa et al., 2016). Psychotherapy is an approach that occupational therapists use in their practice in mental health to achieve these aims (Moll et al., 2022, 2013). Recent changes to legislation across Canada and other jurisdictions, however, have changed the policy and regulatory landscape for occupational therapy in mental health practice regarding psychotherapy in Canada, and there is a need to develop research and practice to support occupational therapists who are practicing in this area.

### Occupational Therapists’ use of Psychotherapy in Mental Health

Occupational therapy was historically a distinct mental health profession originating in the moral treatment movement, a movement aimed at providing dignified living conditions for individuals experiencing mental illness (Friedland, 2011). The profession was based on the belief that there was an important relationship between meaningful time use and mental well-being, and this belief remains central to the philosophy and practice of occupational therapy in the present day (Sedgewick et al., 2007). Though the scope of occupational therapy has since expanded to include physical and cognitive rehabilitation and most recently “social occupational therapy” (Malfitano et al., 2014), mental health practice continues to be a core part of our profession. Occupational therapy in mental health has continued to evolve as a distinct area of practice for occupational therapists, and one that has developed in the context of an ever-changing health care system (Krupa et al., 2016). Occupational therapists offer a range of mental health interventions in services ranging from inpatient and community mental health teams (Moroz et al., 2020) and primary care (Donnelly et al., 2016). While occupational therapists use a range of therapeutic strategies including independent living skills training, developing routines, and addressing barriers to engagement in meaningful activity, psychotherapy is a key approach that is frequently integrated into occupational therapy practice (Moll et al., 2013; COTO, 2018).

Health professionals that comprise Canada's mental health system are increasingly diverse, and include social workers, nurses, psychologists, psychiatrists, occupational therapists, and others (Moroz et al., 2020). These professionals provide support in public, private, and community sectors, and many offer psychotherapy as part of their practice. In recent years, recognition that psychotherapy has been largely underfunded in the public mental health system has led to health professionals and researchers imposing increased pressure on policy makers to fund these services to improve equitable access for all Canadians (MHCC, 2017; Moroz et al., 2020). This has led to the creation of publicly funded psychotherapy including the Ontario Structured Psychotherapy Program, a program offering short-term CBT to individuals in the community living with anxiety and depression (Ontario Health, 2021). It is anticipated that such publicly funded services will continue to become more available across Canada to meet the needs of underserved groups in the future. Expansion of such services will demand a large workforce of health professionals who are competent in the delivery of psychotherapy, including occupational therapists. This represents an opportunity and a challenge for the profession as it evolves alongside an ever-changing service and policy landscape.

### What is Psychotherapy and how Have Occupational Therapists Been Involved?

Psychotherapy is a relatively new phenomenon that emerged in Western societies in the nineteenth century, with historical roots in shamanism and folk medicine (Rieken, 2015). It is focused on helping individuals to gain insight into the state of their mental health and to develop coping strategies that enable them to manage optimally in their daily lives (Nardone & Salvini, 2019). Occupational therapy has long been written about and been involved in the use of psychotherapeutic approaches. Early interventions included the use of art and craft-based strategies primarily informed by a psychodynamic lens (Napoli & Gold, 1947), evolving into more modern uses of solution-focused, and cognitive-behavioural strategies in contemporary practice (Moll et al., 2013). In early literature about occupational therapy in mental health, the profession itself was described *as* a form of psychotherapy, with scholars predicting that its use will become increasingly important over time (Haviland, 1927):

As the mental factor in physical disease receives increasing attention, it necessarily follows that occupational therapy as primarily a form of psychotherapy will be adopted in increasing measure as part of the therapeutic attack upon such diseases. Nonetheless, because it is primarily a form of psychotherapy, it will still find its largest field of usefulness in the treatment of nervous and mental disorders. (p. 431) (Haviland, 1927)

In contemporary practice, occupational therapists frequently regard the use of psychotherapy as an important intervention strategy, with many identifying it as integral to their therapeutic approach (Moll et al., 2022, 2013). Recent changes in the regulatory landscape in Canada, however, have influenced how and whether occupational therapists deliver psychotherapy, and whether it is seen to be within the profession's scope of practice.

### Legislative Changes Influencing Occupational Therapists’ use of Psychotherapy in Canada

Until recently, psychotherapy has been an unregulated therapeutic approach in Canada. Concerns about risks for vulnerable persons, however, have led to increasing regulation across the country and beyond (CCPA, 2021). At the time of preparing this article, psychotherapy regulation has been initiated in all Canadian provinces, with regulation having been fully implemented in Ontario, Quebec, New Brunswick, and Nova Scotia, with Alberta soon to follow (CCPA, 2021). While regulation has opened opportunities for occupational therapists to practice psychotherapy with confidence, it has also limited the practice of some occupational therapists depending on provincial regulatory processes. In Ontario, for instance, psychotherapy was proclaimed a controlled act in 2018 (Government of Ontario, 2021). Occupational therapists were explicitly identified in the Ontario legislation as one of five professions that are legally entitled to deliver psychotherapy (Government of Ontario, 2021). In contrast, only two professions were named in similar legislation in Quebec (medical doctors and psychologists), thereby requiring any professional who is not a medical doctor or psychologist to register to obtain a permit from the Order of Psychologists of Quebec to practice psychotherapy legally in the province (LegisQuebec, 2012). New Brunswick and Nova Scotia have both opted for a competency-based model in which health and social care professionals providing psychotherapy need to supply evidence of having achieved educational and supervisory requirements to register with colleges that regulate psychotherapy in these provinces regardless of professional background (Brunswick, 2017; Nova Scotia Legislature, 2008). While Alberta has not yet proclaimed the regulation of psychotherapy in the province, or formally protected the title “psychotherapist,” the Alberta College of Occupational Therapists openly recognizes the use of psychotherapy by occupational therapists and has encouraged their membership to continue using this title while the regulation process continues (ACOT, 2019).

### Defining Psychotherapy: Is it a Part of OT Practice or not?

Understanding how psychotherapy is defined in a range of jurisdictions has become a legal issue for occupational therapists and other professionals that can influence how occupational therapists *can* practice within a mental health context. The legal definitions provided by the Provinces in Canada that have fully regulated psychotherapy (Ontario, Quebec, New Brunswick, and Nova Scotia) determine what is defined as psychotherapy and if occupational therapists are legally entitled to provide it. In Ontario, the definition of psychotherapy used by the Province of Ontario is as follows:

Psychotherapy involves communication between a client and health provider. This communication helps the client find relief from mental health concerns, find solutions to problems in their life, and change the ways of thinking and acting that are preventing them from working productively, functioning in daily living, and enjoying personal relationships. Psychotherapy could be complemented by other therapies (such as medication and counselling). [Health Professions Regulatory Advisory Council (HPRAC), 2017]

Such a definition would include much of occupational therapy practice in mental health, and includes the importance of occupational outcomes including employment and function in daily life. In the Province of Quebec, however, the definition of psychotherapy explicitly excludes what would typically be considered occupational therapy practice in mental health:

Psychotherapy is psychological treatment for a mental disorder, behavioural disturbance or other problem resulting in psychological suffering or distress, and has as its purpose to foster significant changes in the client's cognitive, emotional or behavioural functioning, his interpersonal relations, his personality or his health. Such treatment goes beyond help aimed at dealing with everyday difficulties and beyond a support or counselling role.

The following interventions are not psychotherapy, but are related to it: accompaniment, support intervention, conjugal and family intervention, psychological education, rehabilitation, clinical follow-up, coaching, and crisis intervention. (Order of Psychologists of Quebec, 2020)

How psychotherapy is defined and how it applies to practice has been the subject of debate among health professionals and will likely persist for some time. While this debate continues, occupational therapists will need to prepare for ongoing regulatory changes that may influence their practice by developing a body of literature that can inform occupational therapy practice and help the profession to further develop an identity in this realm.

### The Current Study

There is a need to understand the state of existing occupational therapy literature on psychotherapy for occupational therapists to develop and maintain a body of evidence pertaining to the contribution that the profession does and can make in this area. We conducted this study to amalgamate existing literature to inform future research efforts, health care policy and practice, and in the interest of building upon occupational therapists’ professional identity in this realm. The research question that we used to guide this paper is: What is the scope of existing literature on psychotherapy written by occupational therapists and/or pertaining to occupational therapy research or practice?

## Method

We conducted a scoping review using the method outlined by Arksey and O’Malley (Arksey & O’Malley, 2005) following Preferred Reporting Items for Systematic Reviews and Meta-Analyses Scoping Review (PRISMA-ScR) guidelines (Tricco et al., 2018). When referring to psychotherapy in this study, we used the definition of psychotherapy articulated by the College of Occupational Therapists of Ontario:

Psychotherapy refers to planned and structured interventions aimed at influencing behaviour and function, by psychotherapeutic means. Psychotherapy is delivered through a therapeutic relationship to change an individual's disorder of thought, cognition, mood, emotional patterns, perception, or memory that may impair the individual's judgement, insight, behaviour, communication, or social functioning as it relates to the performance of daily activities. (COTO, 2018)

### Search Strategy

We developed a search strategy in collaboration with an academic research librarian (RI), which was deployed in May 2019, and updated in December 2020. Following PRISMA guidelines (Moher et al., 2009), we searched 8 databases: Medline, AMED, CINAHL, EMBASE, PsycINFO, Cochrane Database of Systematic Reviews, Sociological Abstracts, and ProQuest Dissertations & Theses. We translated the search strategies using each database platform's command language, controlled vocabulary, and appropriate search fields. Search terms related to the concepts of occupational therapy (e.g., occupation*) and psychotherapy (e.g., psychotherapy*, CBT, dialectical behaviour therapy [DBT]) were combined with a Boolean “AND.” In addition, we hand searched the reference lists of all included articles to identify any not captured using our search strategy. A sample of our Medline search is provided in online Supplemental material.

### Article Selection

We uploaded all citations into Covidence™ (Veritas Health Innovation, 2016), a cloud-based software program used to organize abstracts and assist with collaborative review and analysis. Several members of our research team acted as two independent raters (CM, MM, KM, SA, NK, and AC) and assessed the eligibility of each article for inclusion by conducting a title and abstract screen, followed by a review of full-text articles. At each phase, reviewers compared articles against a set of inclusion and exclusion criteria that had been agreed upon by all authors. These criteria are presented in Table 1. Conflicts that emerged during this process were resolved through discussion and consensus. While we excluded articles written in languages other than English, we compiled a list of foreign language articles with abstracts written in English that met our inclusion criteria based on the abstract alone. This list of articles is provided in online Supplemental material.

**Table 1.** Inclusion and Exclusion Criteria.

Inclusion criteria	
1. Articles pertaining to the use of psychotherapy by occupational therapists were published in an occupational therapy journal, or included an author who identified as an occupational therapist.	
2. Dissertations and theses.	
3. Articles published in all years.	
4. Articles pertained to all age groups.	

Exclusion criteria	
1. Non-peer-reviewed sources (e.g., book reviews, books, anecdotal reports, and commentary).	
2. Conference abstracts.	
3. Articles exploring mental health approaches that were inconsistent with the definition of psychotherapy used in this study (see above).	
4. Articles that explored interventions that did not involve a component of discussion and psychological processing.	
5. Articles exploring solely psychoeducational or independent living skills training.	
6. Articles written in languages other than English.	

### Data Extraction

Using Microsoft Excel, four members of our team extracted descriptive information from included studies (MM, KM, SA, and AC). Specifically, we extracted the following information: study design; publication discipline; journal name; author/sample country; year of publication; lifespan focus (i.e., children, youth, adult, and older adult); and intervention format (i.e., individual vs. group).

### Narrative Synthesis

We uploaded all included articles to Dedoose©, a cloud-based qualitative data management program (SocioCultural Research Consultants, 2018), and conducted an inductive content analysis of included studies using processes described by Graneheim and Lundman (Graneheim & Lundman, 2004). In using this approach, all members of our research team coded relevant statements pertaining to the use of psychotherapy by occupational therapists in included articles. These statements were subsequently organized into themes and refined through discussion and consensus.

## Findings

**Figure 1.**
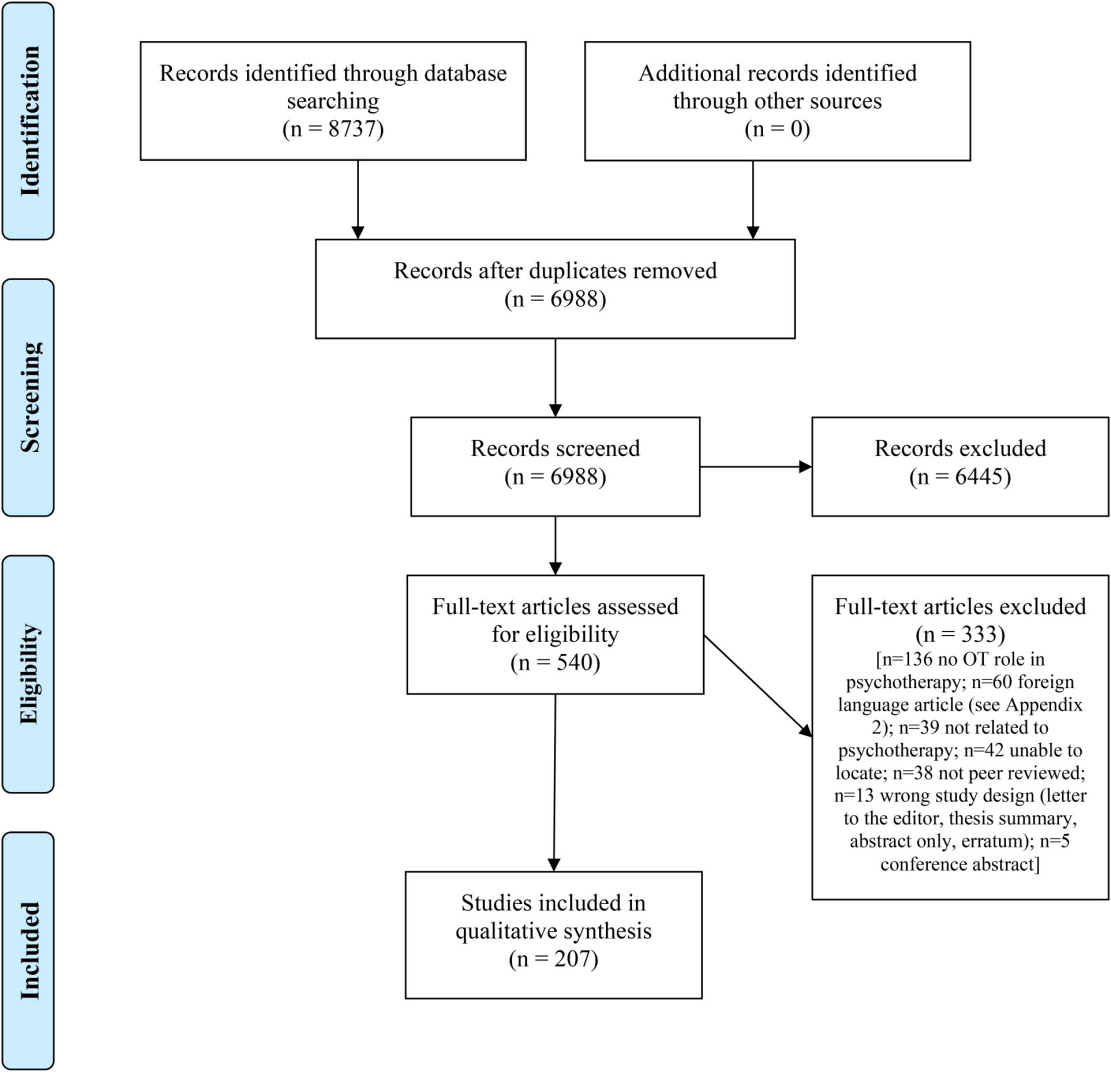
Preferred Reporting Items for Systematic Reviews and Meta-Analyses (PRISMA) flow diagram.

Our search yielded 6,988 records following the removal of duplicates. We eliminated 6,445 during the title and abstract screening, leaving 540 articles that were subjected to full-text review. A total of 207 articles met the criteria for inclusion. A PRISMA flow diagram detailing this process and reasons for exclusion is provided in Figure 1.

*Study design*: Of the 207 articles included in this review, 98 (47.3%) were non-empirical. Details related to study design are provided in Table 2.

**Table 2.** Characteristics of Included Studies (n = 207).

Study Characteristics	n (%)
Year of Publication	
Pre-1950	3 (1.5)
1951–1970	18 (8.7)
1971–1990	56 (27.1)
1991–2010	65 (31.4)
2011–2020	65 (31.4)
	
Lifespan Focus	
Children	21 (10.1)
Children/Youth	5 (2.4)
Children/Adults	5 (2.4)
Youth	4 (1.9)
Youth/Adults	4 (1.9)
Adults	67 (32.4)
Adults/Older Adults	8 (3.9)
Older Adults	17 (8.2)
Not specified/Not applicable	77 (37.2)
	
Intervention Format	
Individual	31 (15.0)
Individual/Group	35 (16.9)
Individual/Community	1 (0.5)
Individual/Family	2 (1.0)
Individual/Family/Group	2 (1.0)
Group	58 (28.0)
Group/Community	1 (0.5)
Family	2 (1.0)
Community	3 (1.4)
Not specified/Not applicable	73 (35.3)
	
Author Country	
United States of America (USA)	85 (41.1)
United Kingdom (UK)	60 (29.0)
Canada	15 (7.2)
Australia	15 (7.2)
Sweden	12 (5.8)
Northern Ireland	3 (1.4)
South Africa	3 (1.4)
Germany	2 (1.0)
The Netherlands	2 (1.0)
Brazil	1 (0.5)
Hong Kong	1 (0.5)
Iran	1 (0.5)
Italy	1 (0.5)
Malta	1 (0.5)
New Zealand	1 (0.5)
Norway	1 (0.5)
Poland	1 (0.5)
Spain	1 (0.5)
Switzerland	1 (0.5)
Turkey	1 (0.5)

*Note*: Percentage sums do not all equal 100 due to rounding.

*Description of included studies*: Studies represented in this review spanned from 1927 to 2020. Details regarding year of publication, lifespan focus, intervention format (individual/group), and author/sample country are provided in Table 3.

**Table 3.** Design of Included Studies (n = 207).

Characteristic	
Study Design	n (%)
Non-empirical	98 (47.3)
Narrative	79 (38.2)
Systematic	15 (7.2)
Scoping	2 (1.0)
Evidence-based review	1 (0.5)
Integrative/Systemic	1 (0.5)
Qualitative	61 (29.5)
Case study	42 (20.3)
General qualitative	9 (4.3)
Qualitative survey	3 (1.4)
Grounded theory	3 (1.4)
Constructivist	1 (0.5)
Case series design	1 (0.5)
Observational	1 (0.5)
Participatory	1 (0.5)
Quantitative	47 (22.7)
Quasi-experimental	19 (9.2)
Randomized control trial	10 (4.8)
Quantitative survey	9 (4.4)
Cross-sectional	4 (1.9)
Longitudinal	2 (1.0)
Delphi	1 (0.5)
Quantitative case study	1 (0.5)
Mixed methods	2 (1.0)
Quasi-experimental (with integrated qualitative methods)	2 (1.0)

*Note*: Percentage sums do not all equal 100 due to rounding.

*Journal of publication*: More than half of the included articles were published in occupational therapy journals. Details regarding journal of publication are provided in Table 4.

**Table 4.** Journal of Publication of Included Studies (n = 207).

Journal Name and Discipline	n (%)
Occupational therapy	138 (66.7)
British Journal of Occupational Therapy	48 (23.2)
American Journal of Occupational Therapy	34 (16.4)
Occupational Therapy in Mental Health	19 (9.2)
Australian Occupational Therapy Journal	11 (5.3)
Occupational Therapy International	7 (3.4)
Canadian Journal of Occupational Therapy	6 (2.9)
Occupational Therapy in Health Care	6 (2.9)
Scandinavian Journal of Occupational Therapy	4 (1.9)
South African Journal of Occupational Therapy	2 (1.0)
OTJR: Occupation, Participation and Health	1 (0.5)
	
Other disciplines	23 (11.1)
American Journal of Psychiatry (Psychiatry)	2 (1.0)
BMC Psychology (Psychology)	2 (1.0)
A.M.A. Archives of Neurology & Psychiatry (Medicine)	1 (0.5)
American Journal of Physical Medicine	1 (0.5)
Annals of the Rheumatic Diseases (Physical Medicine)	1 (0.5)
BMJ Open (Medicine)	1 (0.5)
British Journal of General Practice (Medicine)	1 (0.5)
British Journal of Nursing (Nursing)	1 (0.5)
British Review of Bulimia & Anorexia Nervosa (Psychiatry)	1 (0.5)
Canadian Art Therapy Association Journal (Art Therapy)	1 (0.5)
Children Psychiatry Quarterly (Psychiatry)	1 (0.5)
Current Opinion in Psychiatry (Psychiatry)	1 (0.5)
International Journal of Clinical and Health Psychology (Psychology)	1 (0.5)
International Journal of Geriatric Psychiatry (Psychiatry)	1 (0.5)
International Journal of Social Psychiatry (Psychiatry)	1 (0.5)
Irish Journal of Psychological Medicine (Psychiatry)	1 (0.5)
Nordic Journal of Psychiatry (Psychiatry)	1 (0.5)
Psychoterapia (Psychology and Psychiatry)	1 (0.5)
Psychotherapy: Theory, Research & Practice (Psychology)	1 (0.5)
Rehabilitation Psychology (Psychology)	1 (0.5)
Saudi Medical Journal (Medicine)	1 (0.5)
	
Multidisciplinary	46 (22.2)
ProQuest Dissertations and Theses	11 (5.3)
Physical & Occupational Therapy in Geriatrics	4 (1.9)
Work	3 (1.4)
British Journal of Physical Medicine	2 (1.0)
British Journal of Therapy and Rehabilitation	2 (1.0)
International Journal of Group Psychotherapy	2 (1.0)
Age and Aging	1 (0.5)
Archives of Rehabilitation	1 (0.5)
BMC Health Services Research	1 (0.5)
Clinical Gerontologist	1 (0.5)
CNS Spectrums	1 (0.5)
Epidemiology and Psychiatric Sciences	1 (0.5)
Group Analysis	1 (0.5)
Groupwork	1 (0.5)
Healthcare	1 (0.5)
Health Care in Later Life	1 (0.5)
International Journal of Mental Health	1 (0.5)
International Journal of MS Care	1 (0.5)
International Journal of Therapy and Rehabilitation	1 (0.5)
Journal of Cancer Care	1 (0.5)
Journal of Infant, Child, and Adolescent Psychotherapy	1 (0.5)
Journal of Mental Health	1 (0.5)
Mental Health Review Journal	1 (0.5)
Musculoskeletal Care	1 (0.5)
Pain Medicine	1 (0.5)
Physical & Occupational Therapy in Pediatrics	1 (0.5)
Psychiatric Services	1 (0.5)
The Spine Journal	1 (0.5)


*Note*: Percentage sums do not all equal 100 due to rounding.

### Narrative Synthesis

**Figure 2.**
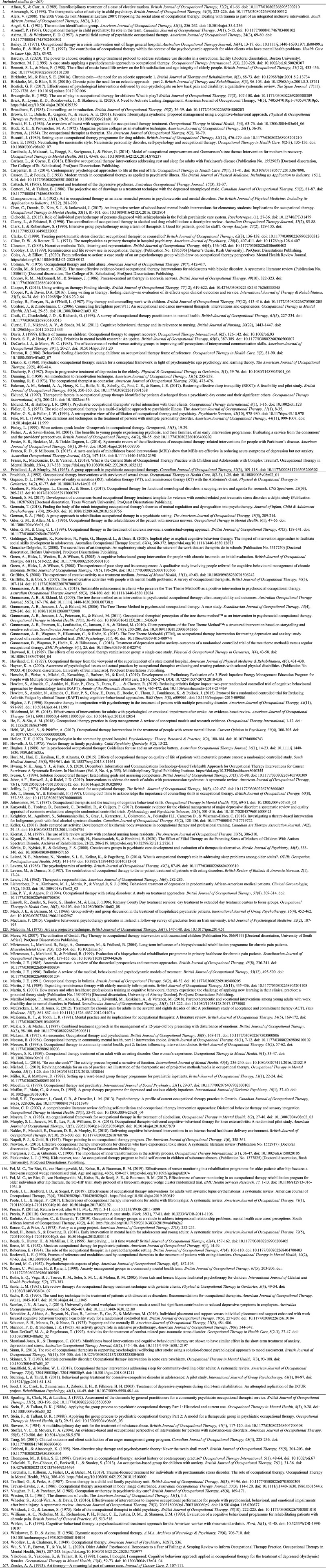
Included studies (n = 207).

We generated four themes through our content analysis of included studies, which are described below. Given the breadth of articles included in this review, we have not provided tables detailing the characteristics of each individual study but have instead provided a numbered list of all included studies in Figure 2. A summary of each theme and sub-theme and articles included in each is provided in Table 6 and can be cross-referenced using Figure 2.

**The psychotherapy practice process.** The most commonly addressed theme in existing literature pertained to how psychotherapy was integrated into occupational therapy practice. We generated four sub-themes that captured the various ways in which this was explored in the occupational therapy literature.

***Intervention modalities.*** A total of 183 (88.4%) of the included studies discussed specific psychotherapy intervention modalities used by occupational therapists. A full summary of the specific studies exploring each psychotherapy intervention modality is summarized in Table 5 and can be cross-referenced using Figure 2. We have provided a narrative synthesis of the three most common psychotherapy approaches explored in the included studies below.

**Table 5.** Psychotherapy Intervention Modalities Explored in Included Studies (n = 207).

Intervention Category	Years of Publication	n^a^	Studies that explored or evaluated this intervention^b^
Activity-oriented approaches (e.g., activity as a medium for psychotherapy, activity-based psychotherapy groups, expressive arts and music as a medium for psychotherapy, play therapy, and the ‘Tree-Theme Method’)	1947–2020	85 (41.7%)	1-2, 6, 8, 15, 20-21, 23-24, 27, 30, 38-40, 42-45, 49-52, 54, 62-63, 69, 75-76, 78, 81-89, 95-96, 101-102, 107, 111-112, 115, 117-118, 121-123, 131, 141-142, 149, 152, 154-155, 157, 159, 161, 163, 165-169, 171, 173, 175-178, 189, 191-193, 195-198, 200, 203-204, 207
Psychodynamic approaches	1947–2018	65 (31.4%)	2-4, 8, 10, 12-13, 20, 22, 27-29, 36, 38, 49, 54, 60-63, 73-75, 82, 92, 96, 108, 111-112, 115, 117-119, 121, 123, 125, 127-129, 131, 138, 141-143, 146, 149, 152, 154, 166-169, 171, 176, 178, 186-189, 191-192, 195-196, 203, 207
Cognitive approaches (e.g., CBT; DBT)	1984–2020	65 (31.4%)	12, 14, 18, 25-26, 31, 47-50, 57, 64-65, 70-72, 75-77, 79-80, 92, 93-95, 98-99, 104, 106, 109, 116, 121-122, 124, 133, 135, 139, 146-151, 153, 156-158, 159-160, 164, 170-171, 175, 178-179, 183-184, 189-190, 193, 199, 201-202, 205-206
Behavioural approaches (including behavioural activation)	1965–2020	14 (6.7%)	1, 7, 14, 16, 36, 53, 68, 72, 99, 120-121, 125, 149, 178
Family psychotherapy(including relationship therapy and family involvement in psychotherapy)	1965–2019	12 (5.8%)	7, 36, 40, 64, 68, 73, 100-101, 108, 129, 162, 164
Trauma-oriented psychotherapy	1986–2019	12 (5.8%)	3, 19, 46, 49, 62, 67, 69, 126, 153, 160, 173, 194
Role-play	1971–2004	10 (4.8%)	4, 19, 36, 45, 51, 138, 176, 189, 195, 200
Reminiscence and life review therapy	1979–2015	9 (4.3%)	8, 11, 38, 70, 90, 97, 113, 132, 172
Exposure therapy	1975–2017	6 (2.9%)	1, 54, 72, 178, 183, 200
Sensory-based approaches	1995–2018	4 (1.9%)	73, 148, 153-154
Gestalt therapy	1981–2007	3 (1.4%)	4, 126, 186
Mindfulness-based psychotherapy (e.g., mindfulness-based cognitive therapy)	1985–2016	3 (1.4%)	17, 66, 199
Narrative therapy	1989–2014	3 (1.4%)	22, 24, 37
Solution-focused therapy	1994–2015	3 (1.4%)	97, 105, 180
Animal-assisted psychotherapy	2005	1 (0.5%)	171
Anxiety management training	1992	1 (0.5%)	185
Filial therapy	2020	1 (0.5%)	114
Group substance use psychotherapy	2020	1 (0.5%)	9
Validation therapy	1996	1 (0.5%)	70

^a^ Number of studies included in each intervention category. Note that some studies were included in more than one category in cases where the authors explored or evaluated more than one intervention modality.

^b^ See Figure 2 to refer to individual studies identified in this table.

Abbreviations: CBT = cognitive behavioural therapy; DBT = dialectical behaviour therapy.

*Activity-oriented approaches*. The use of activity as a means of psychotherapy was explored in 85 (41.7%) of the articles included in this review. The publication dates of these articles ranged from 1947 to 2020. The most common activity identified by the authors of included studies was the use of expressive arts as a means of psychotherapy. Forms of art explored in this body of literature included areas as diverse as the use of finger painting as a projective technique (Napoli & Gold, 1947), puppetry (Schuman et al., 1973), music (Docherty, 1987), and the use of writing as therapy (Cooper & Davis, 2016). One approach developed in recent years, called the “Tree Theme Method” involves painting trees that depict one's past, present, and future as a means of addressing depression and anxiety (Gunnarsson et al., 2015).

*Psychodynamic approaches.* Psychodynamic approaches were explored in 65 (31.4%) of the studies included in this review. The publication dates of these articles ranged from 1947 to 2018. This body of literature explored psychodynamic approaches including projective techniques aimed at uncovering and addressing unconscious conflicts (Fike, 1990), psychodrama and sociodrama (McLean, 1975), object relations therapy (Piergrossi & Gibertoni, 1995), psychoanalysis and psychoanalytic theory (Benetton, 1995; Germain, 2018), and insight-oriented approaches (Moffatt et al., 1995).

*Cognitive approaches*. Cognitive approaches were explored in 65 (31.4%) of the articles included in this review. The publication dates of these articles ranged from 1984 to 2020. Cognitive approaches explored in these studies were overwhelmingly represented by CBT. This included a systematic review focused on approaches used in early intervention with youth including CBT (Read et al., 2018), use of CBT with persons living with physical disabilities such as rheumatoid arthritis (Hewlett et al., 2019), and the use of CBT in individual and group contexts (Johnsonton, 1987). DBT was also explored within this body of literature and included an exploration of the use of DBT with persons living with borderline personality disorder who were engaged in non-suicidal self-injury (Moro, 2007). One study explored how activity could be used to structure the use of DBT with children (Chan et al., 2017).

***Acquiring psychotherapy skills.*** The ways in which psychotherapy skills are acquired by occupational therapists were explored in 37 (18.1%) of the articles included in this review. Some of the authors of included studies identified that occupational therapists begin to incorporate psychotherapy into their practice when working in mental health because the nature of their work simply demands the acquisition of these skills (McLean, 1975). Once occupational therapists recognize the need to develop these skills, authors strongly emphasized the need for adequate training that includes supervision to develop psychotherapy competencies over the course of one's career (Moll et al., 2013).

**Table 6.** Themes Explored in the Occupational Therapy Literature on Psychotherapy (n = 207).

Theme	Sub-themes	n^a^	Studies comprising this theme^b^
The psychotherapy practice process	Intervention modalities^c^	−	−
	Acquiring psychotherapy skills	37 (18.1%)	17, 25, 38, 45-47, 62, 69-70, 73, 84, 86, 88, 92, 97, 100, 108, 115, 117, 121, 123, 126, 133, 138, 141, 144, 147, 152, 165, 176, 189-192, 195, 203, 207
	Outcomes of psychotherapy	29 (14.0%)	17, 24, 31-32, 44, 68-69, 79, 95, 106, 110, 119, 127-128, 131-132, 141, 168, 174-175, 177, 189-190, 193, 195, 200-202, 206
	Assessment	6 (2.9%)	8, 72, 112, 126, 131, 191
			
With whom and for whom psychotherapy is provided	Clinical populations for whom occupational therapists can and do provide psychotherapy	57 (27.5%)	1, 5, 12, 19, 22-23, 25, 27, 28, 31, 33, 35, 40, 42, 44, 46-47, 49, 51, 54, 62, 68-69, 72, 75, 79-80, 91, 95-97, 100, 115, 118, 127-128, 131, 136-137, 139-141, 155, 164, 170, 173-175, 185, 188-190, 196, 199, 201-202, 207
	Psychotherapy delivered by a range of health professionals including occupational therapists.	41 (19.8%)	6, 25, 27, 38, 41, 45-46, 54, 57-58, 60, 62, 69, 74-75, 99, 102, 115, 122, 125, 130, 134, 137, 146, 152, 169, 171, 173-177, 179, 188-190, 193, 197-199, 201
	Unique contributions of occupational therapists to psychotherapy	19 (9.2%)	2, 32, 44, 51, 60, 72, 110, 121, 139-141, 146-147, 167, 174, 178, 187-188, 192
Factors influencing the psychotherapeutic process	Theories that guide occupational therapists’ practice when delivering psychotherapy.	34 (16.4%)	6, 8, 15, 17, 23, 38, 44-45, 52, 56, 58-60, 69, 72, 78, 82, 121, 123, 125-126, 128-129, 140, 145, 149, 153, 165, 168-169, 191-192, 203, 207
	The therapeutic relationship	30 (14.5%)	1-2, 8, 17, 32, 35, 37, 45-46, 62, 68-69, 84, 87-88, 105, 107, 112, 117-119, 123, 131, 153, 165, 169, 191, 195, 200, 206
	The importance of the environment in the delivery of psychotherapy	5 (2.4%)	43, 45, 126, 167, 200
			
Tensions in defining and practicing psychotherapy within the context of occupational therapy	Criticisms of the occupational therapy body of research on psychotherapy	18 (8.7%)	25, 38, 64, 66, 72, 90, 153, 165-166, 170, 189-190, 192-193, 196, 199, 203-204
	The evolving role of occupational therapy in mental health	15 (7.2%)	8, 17, 31, 35, 40, 45-46, 52, 108, 119, 139-140, 147, 185, 188
	Differences between counselling and psychotherapy	15 (7.2%)	8, 17, 29, 31, 35, 40, 45-46, 52, 108, 119, 139-140, 147, 185, 188
	Occupational therapists reluctant to identify their work as psychotherapy	3 (1.5%)	17, 72, 147

^a^ Number of studies included in each intervention category. Note that some studies were included in more than one category in cases where the authors explored or evaluated more than one intervention category.

^b^ See Figure 2 to refer to individual studies identified in this table.

^c^ See Table 4 for a summary of intervention modalities included in this review.

***Outcomes of psychotherapy***. Outcomes that occupational therapists either targeted or aimed to target in their practice was explored in 29 (14.0%) of the articles included in this review. While some authors emphasized coping (Stoffel & Moyers, 2004) and behavioural changes (Chan et al., 2017) as key outcomes of psychotherapy, others focused on occupational outcomes including improvements in workplace performance (Scanlan & Lewis, 2014), returning to valued roles and regaining control within the context of one's occupational life (Meyers, 2010). One author emphasized that there were personal outcomes for occupational therapists as they deliver psychotherapy approaches, including gaining a sense of satisfaction from helping others achieve well-being (Lewis, 1962, p. 283).

***Assessment***. Assessment strategies that occupational therapists use or could use to guide their approach to psychotherapy were explored in 6 (2.9%) articles included in this review. In one of these, the author introduces an approach aimed at improving the daily life functioning of combat veterans with PTSD for use in occupational therapy practice (Gerardi, 2017). In this approach, the author recommends the use of a range of assessment tools well-known in occupational therapy including the Canadian Occupational Performance Measure and others aligning with the Model of Human Occupation (Gerardi, 2017). Scholars also identified that a person's drawings could serve as an important source of assessment material (Keller, 2001), while others highlighted the value of activity analysis to inform future psychotherapy intervention (Banks & Blair, 1997).

**With whom and for whom psychotherapy is provided**. The authors of articles included in this review described the professionals with whom occupational therapists collaborate in the delivery of psychotherapy, and the range of clinical and demographic populations for whom occupational therapists offer psychotherapy services. We generated three sub-themes that capture the ways in which these issues were explored.

***Clinical populations for whom occupational therapists can and do provide psychotherapy.*** The range of clinical populations for whom occupational therapists provide psychotherapy was identified in 57 (27.5%) articles included in this review. These articles explored the use of psychotherapy by occupational therapists in the management of trauma (Cordero & Zimbelmann, 2006), chronic pain (Martensson et al., 2004), body dysmorphia (Meyers, 2010), anxiety (Rosier et al., 2016), substance use (Stoffel & Moyers, 2004), depression (Cooper & Davis, 2016), insomnia (Green et al., 2008) and in the management of stress associated with anger (Tang, 2001).

***Psychotherapy delivered by a range of health professionals including occupational therapists.*** Psychotherapy as an approach delivered by a range of health professionals, including occupational therapists, was described in 41 (19.8%) articles included in this review. Some authors acknowledged the role of occupational therapists in the delivery of psychotherapy within the context of interdisciplinary teams (Wheeler et al., 2016), while recognizing that occupational therapists have the potential to deepen their skills in so doing (Copley et al., 1987). Authors of these studies explored the use of psychotherapy by other professionals as a component of broader interventions in which occupational therapists are involved (Schneider et al., 2016). Others argued that while the authors of some articles exploring psychotherapy do not discuss the involvement of occupational therapists, the use of psychotherapy in these approaches is consistent with the scope of occupational therapy as identified by existing practice frameworks (Conlin & Lorinser, 2012).

***Unique contributions of occupational therapists to psychotherapy***. The unique contributions that occupational therapists can offer to the practice of psychotherapy were identified in 19 (9.2%) articles included in this review. Authors identified that the focus of occupational therapy on activity, and occupational therapists’ deep knowledge of how activity can be used therapeutically places them in a unique and important position to deliver psychotherapy that is activity-based (Ainscough, 1998; Short-Degraff & Engelmann, 1992). Others suggested that existing psychotherapy approaches could be adapted by occupational therapists to be activity-based as a way of aligning more closely with the occupational focus of the profession (Stein & Tallant, 1988). One scholar noted that the contribution of occupational therapists in the realm of psychotherapy is unknown and emphasized the importance of measuring outcomes that would enable occupational therapists to identify the profession's specific contribution (Wheeler et al., 2016).

**Factors influencing the psychotherapeutic process**. Authors of the studies included in this review explored the various factors that influence the psychotherapeutic process. We generated three sub-themes that characterize this overall theme.

***Theories that guide occupational therapists’ practice of psychotherapy***. Theory as a guide for occupational therapists as they practice psychotherapy was explored in 34 (16.4%) articles included in this review. Theories that were most commonly discussed as influential in occupational therapists’ use of psychotherapy and more generally included humanistic (Broadbent, 1985) and psychodynamic theories (Eklund, 1997). There is a clear trend over time in which scholars were quite committed to utilizing the psychodynamic approach to inform how they supported individuals living with mental illness (Copley et al., 1987; Wittkower & Azima, 1958). This began to erode in the 1980s when scholars began to recommend the use of more humanistic approaches that acknowledged the expertise that service users bring to the therapeutic encounter (Thompson & Blair, 1998).

**The therapeutic relationship**. The critical importance of the therapeutic relationship in the delivery of psychotherapy by occupational therapists was explored in 30 (14.5%) articles included in this review. Many authors of included studies simply highlighted how the relationship between occupational therapists and service users was incredibly valuable in the delivery of psychotherapy (Cordero & Zimbelmann, 2006; Keller, 2001). Others emphasized the importance of understanding the concepts of transference and countertransference when offering psychotherapy interventions to help occupational therapists to appreciate the extent to which individuals’ unconscious lives might influence the psychotherapy process, and vice versa (Banks & Blair, 1997).

**The importance of the environment in the delivery of psychotherapy**. The importance of how the environment in which psychotherapy is delivered can influence the psychotherapeutic process was highlighted in 5 (2.4%) of the articles included in this review. Authors emphasized the value of providing a stable social context within therapy sessions that would enhance the impact of therapy, and counteract experiences of instability that might contribute to psychological distress (Copley et al., 1987). Others identified that the therapeutic context must feel emotionally safe for individuals and groups to explore psychological issues, and emphasized the importance of cultivating emotional safety (Maree, 2007).

**Tensions in defining and practicing psychotherapy within the context of occupational therapy**: Tensions in the occupational therapy literature related to defining and practicing psychotherapy were explored in included studies. We generated four sub-themes that summarize this body of literature.

***Criticisms of occupational therapy research on psychotherapy***. A critical perspective of occupational therapy research on psychotherapy was offered in 18 (8.7%) articles included in this review. Authors encouraged researchers to further develop this body of evidence, which was seen to be lacking. Early articles identify the common assumption that the therapeutic use of activity for improving mental health is an effective approach, yet recognize there was little empirical evidence to suggest that this was the case at the time (Wittkower & Azima, 1958). Authors of more recent articles implore researchers to conduct studies on psychotherapy that are specific to the realities of occupational therapy practice as the lack of empirical research in our own profession means that we must draw from the research published in other disciplines to support our use of specific psychotherapy modalities (Newton, 2013; Stoffel & Moyers, 2004).

***The evolving role of occupational therapy in mental health***. Occupational therapists’ use of psychotherapy within the context of an evolving role in mental health was described in 15 (7.2%) articles included in this review. The role of occupational therapists in the delivery of psychotherapy has long been acknowledged within this body of literature, with early articles identifying ways of integrating psychotherapy within an occupational therapy group program (Woolley & Chalmers, 1949), and others calling for the increased use of psychotherapy by occupational therapists working in mental health (Lewis, 1962). In the 1980s, the authors identified that occupational therapists were taking an increasingly independent role in the use of activity-based approaches including art, drama and crafts, and non-activity-based approaches including relaxation therapy and group psychotherapy approaches (Fike, 1990).

***Differences between counselling and psychotherapy***. In 15 (7.2%) articles included in this review, authors contrasted the terms “counselling” and “psychotherapy,” emphasizing the importance of making this distinction in occupational therapy practice. For some scholars, the depth of therapy and the setting in which it was performed determined whether an occupational therapist's approach should be called “psychotherapy.” “Counselling”, on the other hand, referred to the use of approaches in non-medical settings with individuals without a formal diagnosis (Broadbent, 1985). For others, “psychotherapy” was seen to be restricted to occupational therapists who had acquired skills through a rigorous training program involving close supervision by another professional well versed in its practice, whereas counselling was framed as less formal (Lewis, 1962).

***Occupational therapists as reluctant to identify their work as psychotherapy***. The reluctance of occupational therapists to identify their practice as psychotherapy even when their approaches are consistent with known psychotherapy definitions was explored in 3 (1.5%) articles included in this review. In one study, two-thirds of occupational therapists participating in a survey identified that psychotherapy was an essential part of their role, yet 14% identified that their use of psychotherapy was peripheral to their practice of occupational therapy (Moll et al., 2013), a finding reiterated by Gerardi (Gerardi, 2017). In an earlier article, one author encourages occupational therapists to develop skills in psychotherapy, and implies that this is an adjunct skill needed for work in mental health (Broadbent, 1985).

## Discussion

This scoping review represents the first comprehensive synthesis of occupational therapy literature on the topic of psychotherapy, and one that we aimed to conduct to support practice and direct future research in this area. This is an important endeavour given the rapidly evolving practice and policy context related to the regulation of psychotherapy across the provinces and territories in Canada, and the consequent need for a body of evidence that supports occupational therapy practice and the identity of the profession in a mental health context. In this review, we uncovered 207 articles spanning 93 years, demonstrating that occupational therapy researchers and practitioners have been exploring psychotherapy related to the profession for nearly a century. The number of publications grew over time, with over 60% published in the past 30 years. Almost half of these articles were non-empirical, with nearly 40% representing narrative literature reviews. This reveals a need for future empirical study by occupational therapy researchers and an opportunity for developing and evaluating psychotherapy approaches that reflect the context and values of occupational therapy practice.

Our finding that 47.3% of the included studies were non-empirical demonstrates that while occupational therapists have been thinking about and contributing to the literature on psychotherapy for nearly a century, that much of this activity has been limited to commentary on psychotherapy approaches as they relate to occupational therapy practice. Of the 53.2% of studies that were empirical, 20.3% were case studies. Only 15% of the included articles were designed to evaluate a psychotherapy intervention using quasi-experimental and randomized control trial designs. The large number of non-empirical studies and the limited number of studies designed to evaluate an intervention suggests that while this body of literature boasts 207 articles, it remains in an early stage of development.

A majority of the articles (88.4%) included in this review explored psychotherapy intervention modalities with activity-based, psychodynamic, and cognitive approaches being the most commonly explored. Activity-oriented approaches represented nearly half of the psychotherapy interventions explored in these articles, which is unsurprising given the focus of the profession on engagement in and performance of meaningful activity (Townsend & Polatajko, 2013). Not only did the articles included in this review explore activity-oriented approaches, but also identified how the profession's expertise in occupation may offer an important lens and an opportunity for occupational therapy to make a unique contribution in the realm of psychotherapy (Ainscough, 1998; Short-Degraff & Engelmann, 1992). While activity-based psychotherapy approaches exist, including art, drama, writing, and dance therapies, there is an opportunity for occupational therapists to build on these approaches by developing novel interventions informed by the profession's expertise. One such approach, the Tree Theme Method, represents an example of an intervention that has been developed specifically by occupational therapy researchers using an occupational lens (Gunnarsson et al., 2015). While framed as a “psychosocial intervention” (Gunnarsson et al., 2015) by the authors of this approach, the method of asking an individual to engage in introspection by reflecting on their past, present, and future through the medium of painting trees is certainly consistent with the definition of psychotherapy used in this review (COTO, 2018). Further, authors of the articles included in this review emphasized that occupational therapists can demonstrate their unique contribution to occupational therapy by measuring occupational outcomes such as participation and function in meaningful activity (Wheeler et al., 2016). By developing novel approaches, and measuring their effectiveness on occupational outcomes, there is an opportunity to build upon and deepen the contributions that occupational therapy can make in the realm of psychotherapy both within and outside of the profession. It should be noted that art, music, play, and dance therapies are regularly considered to be psychotherapy modalities (Canadian Counselling and Psychotherapy Association, 2021), and occupational therapists who use expressive activities with the aim of improving the mental health of individuals with whom they work should recognize the implications of using these approaches in practice.

Psychodynamic and cognitive approaches were equally explored in the articles included in this review with 31.4% of included studies discussing each. An increase in the number of articles exploring cognitive modalities published in recent years, and a decrease in articles exploring psychodynamic approaches is consistent with the interdisciplinary literature on psychotherapy where psychodynamic approaches have waned in recent decades while cognitively oriented approaches have been broadly embraced (Cortina, 2010; Krupa et al., 2016). Interdisciplinary scholars, however, warn against this trend given existing evidence indicating that psychodynamic psychotherapy boasts similar effectiveness when compared with non-psychodynamic approaches (Shedler, 2010). Occupational therapy researchers and practitioners are encouraged to consider this reality when selecting training opportunities and designing research aimed at evaluating psychotherapy approaches.

Authors of included studies emphasized the factors that influence the psychotherapeutic process including theory, the therapeutic relationship, and the importance of cultivating an environment promoting the emotional safety of individuals receiving psychotherapy. Occupational therapy's theoretical positioning in humanism has persisted across the history of the discipline's discussion of psychotherapy, a commitment that is not restricted to this body of literature (Brown, 2013; Friedland, 2011; Townsend & Polatajko, 2013). The importance of attending to transference and countertransference in the delivery of psychotherapy is reflective of the influence of the psychodynamic model throughout the history of occupational therapy, and remains relevant in practice today with the College of Occupational Therapists of Ontario highlighting the importance of attending to these factors in the delivery of psychotherapy by occupational therapists (COTO, 2018).

Finally, occupational therapy researchers and practitioners highlighted the reluctance of the profession to declare their work as psychotherapy in this review. This literature suggests that the identity of occupational therapists in the realm of psychotherapy has been contentious for the profession with some scholars calling for increased integration of psychotherapy, and others viewing psychotherapy as a parallel approach or an approach best delivered by other professions (Moll et al., 2013). Surely, this reluctance is likely to persist in the context of provincial legislation determining how and whether occupational therapists can legally deliver psychotherapy in their respective jurisdictions. While this perspective represented a minority of articles included in this review, it does reveal a contention well known anecdotally by occupational therapists who provide psychotherapy as part of their practice.

### Research Implications

The articles included in this review have highlighted the need for further psychotherapy research in occupational therapy as the role of occupational therapists continues to evolve in Canada. Only 7.2% of included articles were published by Canadian researchers, highlighting the need to develop a body of evidence that reflects the realities of a Canadian context. Authors of included articles emphasized the need to conduct research on psychotherapy from an occupational perspective, as a lack of research reflecting the realities of the discipline results in the need to draw on research in other professions that face different practice realities and areas of focus (Newton, 2013; Stoffel & Moyers, 2004). There is both an opportunity and a need to develop a body of literature that reflects the perspectives and unique contributions that occupational therapists can make in the realm of psychotherapy. This can serve to promote the identity of occupational therapists in the provision of psychotherapy and highlight the unique contribution that the profession can make in this area.

### Practice Implications

The authors of included articles emphasized how occupational therapists frequently learn psychotherapy when practicing mental health because the realities of their practice context demand such skills (McLean, 1975). This is particularly relevant given the changing policy and practice landscape in mental health across Canada. Occupational therapists have long been integrated with publicly funded mental health services across the country. An anticipated increase in publicly funded psychotherapy that has been initiated (Ontario Health, 2021) will likely increase demands on health and social care professionals to develop competence in evidence-based psychotherapy approaches such as CBT and DBT. Remaining relevant in the context of this rapidly changing context means that occupational therapists need to be prepared to develop and maintain psychotherapy skills. This will demand a culture within occupational therapy that can support the acquisition of psychotherapy skills through education and supervision, as well as opportunities for maintaining and deepening competence over time.

Throughout this scoping review, our team had multiple conversations about definitions of psychotherapy and how it is variously defined by different professional groups and in a range of jurisdictions. This challenged our team to compare these definitions against articles that described a range of intervention approaches and included those that were consistent with the definition of psychotherapy used to guide this review. We recognize that occupational therapists have long used activity-based approaches that are clearly aligned with existing definitions of psychotherapy including play and expressive arts therapies, a claim that has been substantiated by the findings of this review. Talk-based therapies, however, are not explicitly linked to occupation, yet appear to be broadly used in practice and explored in the literature that we have synthesized in this paper. We recognize that the use of these talk-based approaches, many of which are supported by evidence, are not apparently activity- or occupation-focused in their orientation. The use of such approaches by occupational therapists may risk diluting the profession's identity as a discipline that holds occupation at its core. Occupational therapists using talk-based approaches are encouraged to clearly link their use of such modalities to occupational performance and/or engagement in their practice. Making this linkage can help occupational therapists to deliver talk-based approaches in a manner that is consistent with the occupational focus of the profession. Further, expansion of psychotherapy programs may not include funding for activity-based forms of psychotherapy, and occupational therapists are cautioned to find ways of using approaches that are funded in a way that maintains the unique occupational contribution that our profession does and can make in this area of practice.

### Limitations

While our search was comprehensive, there is still a possibility that we may have missed articles that were unavailable in the Western University Library databases. Further, we struggled particularly with defining psychotherapy and distinguishing it from more general occupational therapy approaches used with individuals living with mental health challenges. For this reason, there is a possibility that in an effort to include articles corresponding solely to the definition of psychotherapy included in this review, some may have been inadvertently excluded based on terminology or descriptions of psychotherapy in the abstracts and full-text articles that we reviewed. The majority of studies included in this review were published by scholars located in the United States and UK, and in journals representing these contexts. The findings, thus, represent these sociocultural contexts, and should be interpreted in consideration of this reality. Further, racial characteristics were largely unavailable for included studies, and as a result, this information was not captured in this review. Little is known about the extent to which the findings of this study pertain to persons of a range of races, genders, sexual orientations, and other social locations.

## Conclusion

This review demonstrates that occupational therapy has a long history with psychotherapy research and practice that has spanned nearly a century. While the body of literature synthesized in this review included psychodynamic, cognitive, behavioural, and several other modalities, a significant portion of literature included in this review explored activity-based approaches, representing more than 41% of included articles. The focus of this literature on activity-based psychotherapy reveals the expertise that occupational therapists do and can bring to this practice area—both in using approaches that integrate the use of activity, and in targeting function and participation as an outcome. More empirical research demonstrating these concepts, however, is needed. Tensions about whether psychotherapy is considered part or peripheral to occupational therapy practice reveals the discomfort that the profession has grappled with around the inclusion of psychotherapy in practice. Whether occupational therapists acknowledge psychotherapy as a part of their practice, the findings of this review demonstrate that it clearly has a place in the profession's public discourse. Such dialogue is likely to continue in the rapidly changing policy landscape within which the profession is situated in Canada. While these realities continue to unfold, we hope that this review serves to provide a sense of collective pride and enables occupational therapists to embrace their identity as one that includes psychotherapy as part of our past, present, and future.

## Key messages

- Occupational therapy research and practice has been concerned with psychotherapy for nearly a century
- Recent changes in the regulation of psychotherapy across Canada has drawn attention to the importance of understanding occupational therapists’ involvement in psychotherapy both historically and in the present to inform future research and practice
- Empirical research demonstrating the unique contributions that occupational therapists do and can make in the realm of psychotherapy are needed

## Supplemental Material

sj-docx-1-cjo-10.1177_00084174221102732 - Supplemental material for Psychotherapy Within Occupational Therapy Literature: A Scoping Review Click here for additional data file. Supplemental material, sj-docx-1-cjo-10.1177_00084174221102732 for Psychotherapy Within Occupational Therapy Literature: A Scoping Review by Carrie Anne Marshall, Michelle Murphy, Kristina Marchiori, Suliman Aryobi, Pam Wener, Catherine White, Nadine Larivière, Roxanne Isard, Avneet Chohan, Mary Forhan, Niki Kiepek, Skye Barbic, Victoria Sarunsky and Sandra Moll in Canadian Journal of Occupational Therapy

sj-docx-2-cjo-10.1177_00084174221102732 - Supplemental material for Psychotherapy Within Occupational Therapy Literature: A Scoping Review Click here for additional data file. Supplemental material, sj-docx-2-cjo-10.1177_00084174221102732 for Psychotherapy Within Occupational Therapy Literature: A Scoping Review by Carrie Anne Marshall, Michelle Murphy, Kristina Marchiori, Suliman Aryobi, Pam Wener, Catherine White, Nadine Larivière, Roxanne Isard, Avneet Chohan, Mary Forhan, Niki Kiepek, Skye Barbic, Victoria Sarunsky and Sandra Moll in Canadian Journal of Occupational Therapy
