# Supplementary material for: Psychotherapy Within Occupational Therapy Literature: A Scoping Review
Source: Can J Occup Ther. 2022 Jul 26;89(4):376–94. doi: 10.1177/00084174221102732 (PMC9709557; doi:10.1177/00084174221102732)
Supplement: sj-docx-1-cjo-10.1177_00084174221102732 - Supplemental material for Psychotherapy Within Occupational Therapy Literature: A Scoping Review [file sj-docx-1-cjo-10.1177_00084174221102732.docx]

Appendix 1. Sample Search Strategy – Medline

Database: Ovid MEDLINE(R) ALL <1946 to December 17, 2020>

Search Strategy:

--------------------------------------------------------------------------------

1 occupational therap*.mp. (20455)

2 Psychotherapy/ or psychotherap*.mp. (92058)

3 counsel*.mp. [mp=title, abstract, original title, name of substance word, subject heading word, floating sub-heading word, keyword heading word, organism supplementary concept word, protocol supplementary concept word, rare disease supplementary concept word, unique identifier, synonyms] (137000)

4 Dialectical behavio?ral therapy.mp. (174)

5 DBT.mp. (2721)

6 Mentalization.mp. or Mentalization/ (768)

7 mentalisation.mp. (87)

8 Schema-focused.mp. (75)

9 Brief psychotherapy.mp. or Psychotherapy, Brief/ (3716)

10 Narrative therapy.mp. or Narrative Therapy/ (337)

11 Solution-focused.mp. (408)

12 Trauma based.mp. (253)

13 Trauma informed.mp. (1253)

14 Humanistic therapy.mp. (10)

15 Interpersonal therapy.mp. (348)

16 IPT.mp. (2437)

17 Cognitive behavioural therapy.mp. (4225)

18 Cognitive behavio?ral therapy.mp. or Cognitive Behavioral Therapy/ (31870)

19 CBT.mp. (11628)

20 Cognitive therapy.mp. (3263)

21 Rational emotive behavio?r therapy.mp. (81)

22 REBT.mp. (61)

23 Psychotherapy, Psychodynamic/ or psychodynamic.mp. (5494)

24 Motivational Interviewing/ or motivational interview*.mp. (4921)

25 Mindfulness.mp. or Mindfulness/ (8752)

26 MBCT.mp. (492)

27 MBSR.mp. (693)

28 third wave.mp. (710)

29 (acceptance and commitment therapy).mp. [mp=title, abstract, original title, name of substance word, subject heading word, floating sub-heading word, keyword heading word, organism supplementary concept word, protocol supplementary concept word, rare disease supplementary concept word, unique identifier, synonyms] (1136)

30 Self-compassion therapy.mp. (0)

31 family focused therapy.mp. (59)

32 behavioural activation.mp. (412)

33 behavioral activation.mp. (1501)

34 play therapy.mp. or Play Therapy/ (1300)

35 music therapy.mp. or Music Therapy/ (4461)

36 drama therapy.mp. or Psychodrama/ (1121)

37 Exposure Therapy.mp. (2385)

38 implosive Therapy.mp. or Implosive Therapy/ (1368)

39 integrative psychotherap*.mp. [mp=title, abstract, original title, name of substance word, subject heading word, floating sub-heading word, keyword heading word, organism supplementary concept word, protocol supplementary concept word, rare disease supplementary concept word, unique identifier, synonyms] (97)

40 psychoanaly*.mp. or Psychoanalysis/ (36536)

41 IPSRT.mp. (49)

42 gestalt therapy.mp. or Gestalt Therapy/ (192)

43 TTM.mp. (1692)

44 (interpersonal and social rhythm therapy).mp. [mp=title, abstract, original title, name of substance word, subject heading word, floating sub-heading word, keyword heading word, organism supplementary concept word, protocol supplementary concept word, rare disease supplementary concept word, unique identifier, synonyms] (90)

45 family therapy.mp. or Family Therapy/ (9919)

46 2 or 3 or 4 or 5 or 6 or 7 or 8 or 9 or 10 or 11 or 12 or 13 or 14 or 15 or 16 or 17 or 18 or 19 or 20 or 21 or 22 or 23 or 24 or 25 or 26 or 27 or 28 or 29 or 31 or 32 or 33 or 34 or 35 or 36 or 37 or 38 or 39 or 40 or 41 or 42 or 44 or 45 (312269)

47 1 and 46 (1522)

48 limit 47 to dt=20190501-20201217 (90)

***************************
