# Supplementary material for: Psychotherapy Within Occupational Therapy Literature: A Scoping Review
Source: Can J Occup Ther. 2022 Jul 26;89(4):376–94. doi: 10.1177/00084174221102732 (PMC9709557; doi:10.1177/00084174221102732)
Supplement: sj-docx-2-cjo-10.1177_00084174221102732 - Supplemental material for Psychotherapy Within Occupational Therapy Literature: A Scoping Review [file sj-docx-2-cjo-10.1177_00084174221102732.docx]

Appendix 2. Non-English Language Articles Meeting Inclusion Criteria Based on an English Abstract

1. Acanda Roque, M.C., Gonzalez Valiente, A., & Fiallo Sanza, A. (1990). Psychogeriatrics and psychoballet. *Revista Cubana de Enfermeria, 6*(2), 198-204.
2. Adamovic P. (1989). Day hospitalization program for depressed patients. *Socijalna Psihijatrija, 17*(3), 257-270.
3. Arbousse Bastide, J.C. (1973). Social improvements in a chronic sick ward. II. Psychological aspects. *Nouvelle Presse Medicale,* *2*(20), 1369-1371.
4. Becerra, J.D. (1979). Rehabilitation activities at the Valdes Anciano ward. *Revista del Hospital Psiquiatrico de la Habana, 20*(2), 367-371.
5. Bohlken, J., Burger-Deinerth, E. M., Gratz, T., Hauser, R., Otto, M., Urban, R., & Wilke-Burger, H. (2005). Occupational therapy in psychiatric practice. *Psychoneuro, 31*(1), 42-43. doi: 10.1055/s-2005-863100
6. Boss, M. (1938). Individual treatment preparatory to group occupational therapy in severe cases of chronic schizophrenia. *Schwizer Archiv fur Neurologie, Neurochirurgie und Psychiatrie, 42,* 15-26.
7. Brecic, P., Ostojic, D., Stijacic, D., & Jukic, V. (2013). From occupational therapy and recreation to psychosocial methods of treatment and rehabilitation of psychiatric patients at Vrapce University. *Socijalna Psihijatrija, 41*(3), 174-181.
8. Burkhard, A. (2006). Mindfulness and skill training. *Ergotherapie & Rehabilitation, 45*(5), 16-19.
9. Cerny, J., & Ochrymcuk, L. (1963). Creative products of tin, metal and wire and their use in the treatment of mentally disordered children. *Ceskoslovenska Psychiatrie, 59*(4), 234-240.
10. Coqui, U. (2012). Occupational therapy in a network of multi-generation houses. *Ergotherapie & Rehabilitation, 51*(7), 10-14. doi: 10.2443/skv-s-2012-51020120701
11. Corboz, R. J., & Kohler, R. (1983). About essence and indications of ergotherapy in child psychiatry. *Acta Paedopsychiatrica, 49*(5), 247-250.
12. Dahl, A., & Jorgensen, M.G. (1985). Intensive outpatient milieu therapy and psychotherapy of young schizophrenic patients: Description of the Nordhuset Treatment Center in Helsingor. *Nordisk Psykiatrisk Tidsskrift*, *39*(6), 509-511.
13. Deshaies, M. (1965). The meaning of psychotherapy. *Bulletin de Psychologie, 18*(16/240), 1134-1141.
14. Dietrich, S., Mergl, R., & Rummel-Kluge, C. (2017). From the first symptoms of depression to treatment. When and where are people seeking help? Does stigma play a role? *Psychiatrische Praxis, 44*(08), 461-468. doi: 10.1055/s-0042-113237
15. Donnoli, V. F. (1991). Review of the concept of personality deterioration in schizophrenia. *Acta Psiquiatrica y Psicologica de America Latina, 37*(1), 37-45.
16. Dos Santos, O. (1964). Group occupational therapy. *Jornal Brasileiro de Psiquiatria, 13*(2), 205-218.
17. Emmel, M.L., & Matsukura T.S. (1989). Support group of mothers with children in occupational therapy. *Psicologia: Teoria e Pesquisa, 5*(3), 315-323.
18. Faure, J. (1948). Facts of a neuropsychiatric order observed in the course of a mission of a year and a half in North America. *Journal de Medecine de Bordeaux, 125*(4), 155-162.
19. Filipp, C. (2003). EIKO- parent information concept computer program to support parent and patient counseling. *Ergotherapie & Rehabilitation, 42*(9), 18-20.
20. Frank, B. (1955). Occupational therapy in semi-open care of male nervous and mental patients in Cologne. *Der Offentliche Gesundheitsdienst, 17*(5), 165-169.
21. Gastager, H. (1968). Early and late rehabilitation of psychoses. *Der Nervenarzt, 39*(10), 456-459.
22. Geyer, D., Batra, A., Beutel, M., Funke, W., Gorlich, P., Gunthner, A., … Schmidt, L.G. (2006). AWMF Guideline: Post-acute treatment of alcohol abuse and dependence. *Sucht,* *52*(1), 8-34. doi: 10.1463/2006.01.03
23. Gierig, L., Hulsewiesche, D. (2002). The orientation group: A systemic solution –focused approach including expression centred methods. *Ergotherapie und Rehabilitation, 41*(7), 15-18.
24. Goeppert, H. (1962). Clinical psychotherapy of the neuroses in the framework of the psychiatric hospital. *Der Nervenarzt, 33*(3), 106-111.
25. Gontard, A.L., Lantheaume, S., Martinho, D., & Fernandez, L. (2017). Alzheimer’s disease and self-esteem: Creation of an art-therapy group in an institution for elderly. *NPG Neurologie – Psychiatrie - Geriatrie, 17*(98), 100-108. doi: 10.1016/j.npg.2016.05.005
26. Hadlik, J. (1954). Some theoretical problems of occupational therapy in psychiatric hospitals. *Neurologie a Psychiatrie Ceskoslovenska, 17(*6), 319-326.
27. Hayasaka, T., Kobayashi, O., Kurokawa, Y., Sakai, M., Watarai, M., Itabashi, T., … Kawasoe, Y. (2016). Development of group psychotherapy focused on emotion for substance use disorders: Serigaya Collaboration for Open Heart Project (SCOP). *Nihon Arukoru Yakubutsu Igakkai Zasshi, 51*(3), 203-213.
28. Heredia, L. P. D., & Marziale, M. H. P. (2010). Roles of professionals in drug outpatient care centers, in the city of Bogota, Colombia. *Revista Latino-Americana de Enfermagem, 18,* 573-581. doi: 10.1590/s0104-11692010000700013
29. Hesse, P.W. (2009). Psychology and occupational therapy – what can we learn from each other? *Ergotherapie & Rehabilitation, 48*(9), 19-24.
30. Heyde, W., & von Langsdorff, P. (1983). Rehabilitation of cancer patients including creative therapies. *Die Rehabilitation, 22*(1), 25-27.
31. Higman, P. (2002). Chronic pain and occupational therapy. *Ergotherapie und Rehabilitation, 41*(4), 15-19.
32. Honicke, M. (2011). Acceptance and commitment therapy as a challenging approach for occupational therapists in pain management. *Ergotherapie & Rehabilitation, 50*(7), 28-30.
33. Hoos, B. (1974). Development of a group for creative work in the context of occupational treatment on a psychotherapy ward. *Beschaftigungstherapie und Rehabilitation, 13*(1), 35-37.
34. Horn, M. (2015). When boundaries blur. *Ergotherapie und Rehabilitation, 54*(12), 16-19.
35. Hsin-Tien, L. (1963). The application of psychotherapy in the treatment of chronic schizophrenia. *Acta Psychologica Sinica, 22*(1), 55-64.
36. Janssen, P.L. (1978). To some psychotherapeutic aspects of occupation therapy in a psychiatric hospital. *Psychotherapie und Medizinische Psychologie, 28*(6), 183-193.
37. Jerez, S., Alvarado, L., Paredes, A., Montenegro, A., Montes, C., & Venegas, L. (2001). The University Psychiatric Clinic Personality Disorder Program: A multidisciplinary approach. *Revista Chilena de Neuro-Psiquiatria, 39*(1), 69-76. doi: 10.4067/S0717-92272001000100020
38. Kaloudi, E., Christodoulou, C., Kontaxakis, V., Lykouras, L., & Livaditis, M. (2011). Psychotherapeutic interventions in stereotypies. *Psychiatrike = Psychiatriki, 22*(2), 148-157.
39. Kraus, G. (1949). Something about occupational therapy in psychiatric facilities. *Tijdschrift voor ziekenverpleging, 2*(19), 469-471.
40. Leclerc-Springer, J. (1994). Sprouting roots, growing and matruring. Agricultural occupational therapy as the favoured non-pharmacologic method for treatment and rehabilitation of new chronic patients. *Psychiatrische Praxis, 21*(5), 196-198.
41. Lowenstein, O. (1927). Some experimental and clinical bases for the application of psychotherapy in psychoses with special reference to occupational therapy. *Zeitschrist fur die Gesamte Neurologie und Psychiatrie, 110*, 50-60.
42. Mehle, M. (1966). Results of psychodynamic concepts in occupational therapy. *Ergebnisse der Psychodynamischen Konzeptionen in der Arbeitstherapie*, *23*(4) 327-340.
43. Mirskaia, M.M., Povorinskii, I.A., & Rubinova, R.S. (1960). Experience with the application of occupational therapy within the general complex of therapeutic measures in a closed psychiatric ward. *Zhurnal Nevropatol Psikhiatr Im S S Korsakova, 60,* 624-626.
44. Montrezor, J.B. (2013). Occupational Therapy in the practice of therapeutic groups and workshops with mental health patients. *Cadernos de Terapia Ocupacional da UFSCar, 21*(3), 529-536. doi: 10.4322/cto.2013.055
45. Neto, D.A., & Borges, I.R. (1971). Organization and operation of a day hospital in a private clinic. *Acta Psiquiatrica y Psicologica de America Latina, 17*(1), 33-38.
46. Plana Angles, L. (1994). Psychotic delirious theme in the pictorial expression in psychopathology: Mythologems. (Publication No. C519741) [Universitat de Barcelona (Spain)]. ProQuest Dissertations Publishing.
47. Puertas Hyman, G. (1975). The music in occupational therapy and rehabilitation summary. *Revista del Hospital Psiquiatrico de la Habana, 16*(2), 229-242.
48. Quilelli Correa, R.A. (1964). The musical activity in occupational therapy. *Jornal Brasileiro de Psiquiatria, 13*(2), 239-254.
49. Rihmer, Z., Nemeth, A., Kurimay, T., Perczel-Forintos, D., Purebl, G., & Dome, P. (2017). Recognition, care and prevention of suicidal behaviour in adults. *Psychiatria Hungarica: A Magyar Pszichiatriai Tarsasag tudomanyos folyoirata, 32*(1), 4-40.
50. Rizzo, R. (2014). Fighting depression following strokes – self-management as a method. *Ergotherapie und Rehabilitation, 53*(8), 22-26. doi: 10.2443/skv-s-2014-51020140802
51. Rothenberger, E., Weber-Bruderer, M., & Aegler, B. (2011). Cognitive behavioural therapy and acceptance and commitment therapy in occupational therapy for clients with chronic pain. *Ergoscience, 6*(3), 90-97. doi: 10.2443/skv-s-2011-54020110301
52. Sala, L., Carnart, N., Derval, A., & Rambaud, D. (2018). Preliminary results of mindfulness-based cognitive group therapy (MBCT) in social phobic patients. *Annales Medico-Psychologiques, 176*(5), 495-500. doi: 10.1016/j.amp.2017.06.003
53. Schneller-Reindell, B. (1983). Clinical treatment process of an anorexic patient. *Praxis der Psychotherapie und Psychosomatik, 28*(3), 107-116.
54. Schoene-Adibo, A. (2002). The wayback – The return from war trauma to normalcy: Report on a stay in Sudan in Oct./Nov. 2001. *Ergotherapie und Rehabilitation, 41*(8), 24-28.
55. Selig, D., Schell, B., Scholz, M., Asen, E. (2002). Systemische Ressourcenarbeit mit Familien im ergotherapeutischen Kontext der kinder- und Jugendpsychiatrie. *Ergotherapie und Rehabilitation, 41*(5), 7-13.
56. Stanczak, T., & Adamczyk, K. (1981). The responsibilities of occupational therapists in psychiatric services. *Psychiatria Polska, 15*(4-6), 379-385.
57. Tejero-Morales, S., Cerdena-Macias, I., & Espinosa-Moreno, R. (2015). Psychosocial intervention in borderline personality disorder. *Rehabilitation Psicosocial, 12*(2), 25-31.
58. Van der Linden, P. (1991). Experiences with a form of training therapy in clinical psychotherapy. *Tijdschrift voor Psychotherapie, 17*(1), 3-11.
59. Voelzke, M., & Krueger, S. (2016). Dialectic behavioural therapy. *Ergotherapie und Rehabilitation, 55*(2), 22-26. doi: 10.2433/skv-s-2016-51020160203
60. Zago, F.C., Bredariol, A.C.P., & Paulo de Mesquita, D. (2013). Community therapy application in intervention with adolescents: new strategies for prevention and promotion. *Cadernos de Terapia Ocupacional da UFSCar, 21*(2), 361-371. doi: 10.4322/cto.2013.037
